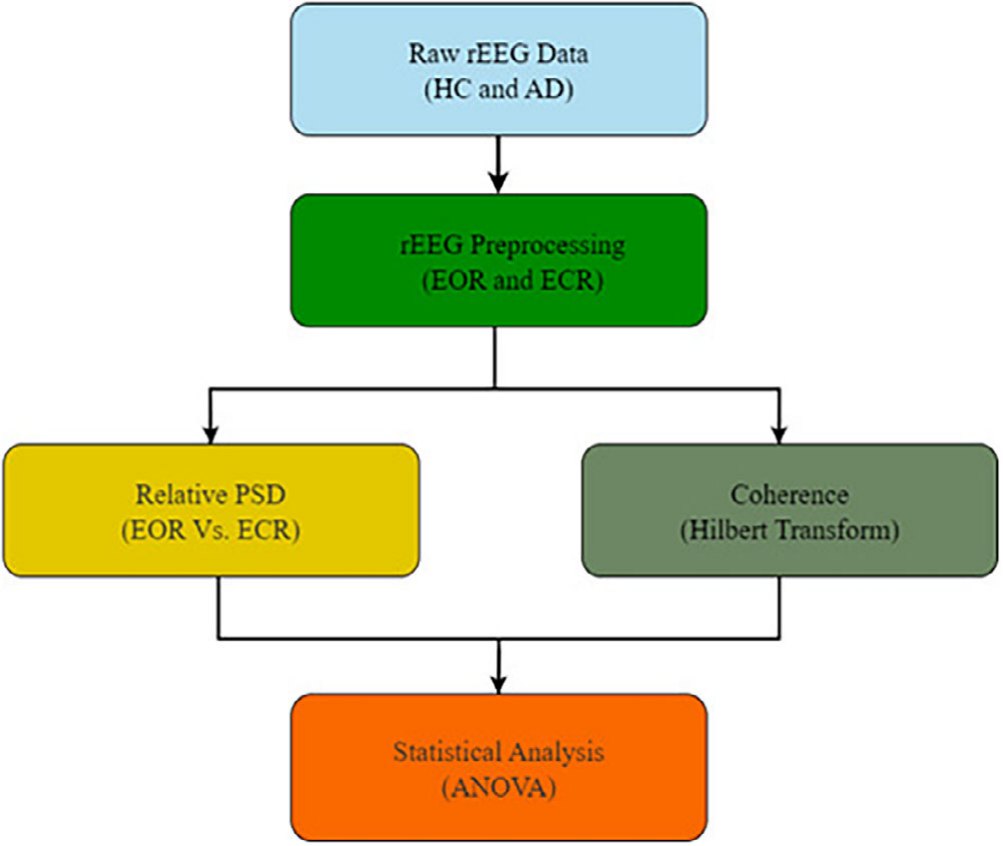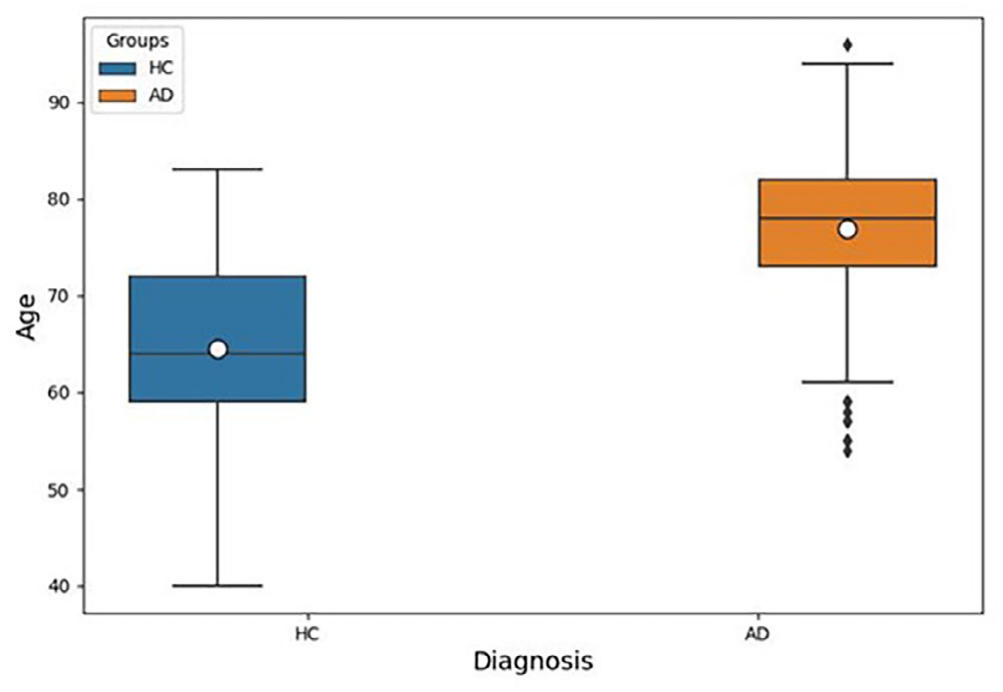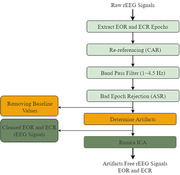# qEEG as Biomarker for Alzheimer’s Disease: Investigating Relative PSD Difference and Coherence Analysis

**DOI:** 10.1002/alz.088587

**Published:** 2025-01-09

**Authors:** Chanda Simfukwe, Young Chul Youn, Seong Soo An

**Affiliations:** ^1^ Chung‐Ang University, Seoul Korea, Republic of (South); ^2^ Department of Neurology, Chung‐Ang University College of Medicine, Seoul Korea, Republic of (South); ^3^ Department of Bionano Technology, Gachon University, Seongnam Korea, Republic of (South)

## Abstract

**Background:**

Electroencephalography (EEG) is a non‐intrusive technique that provides comprehensive insights into the electrical activities of the brain’s cerebral cortex. The brain signals obtained from EEGs can be used as a neuropsychological biomarker to detect different stages of Alzheimer’s disease (AD) through quantitative EEG (qEEG) analysis. This paper investigates the difference in the abnormalities of resting state EEG (rEEG) signals between eyes‐open (EOR) and eyes‐closed (ECR) in AD by analyzing 19‐ scalp electrode EEG signals and making a comparison with healthy controls (HC).

**Method:**

The rEEG data from 534 subjects (ages 40–90) consisting of 269 HC and 265 AD subjects in South Korea were used in this study. The qEEG for EOR and ECR states were performed separately for HC and AD subjects to measure the relative power spectrum density (PSD) and coherence with functional connectivity to evaluate abnormalities. The rEEG data were preprocessed and analyzed using EEGlab and Brainstorm toolboxes in MATLAB R2021a software, and statistical analyses were carried out using ANOVA.

**Result:**

Based on the Welch method, the relative PSD of the EEG EOR and ECR states difference in the AD group showed a significant increase in the delta frequency band of 19 EEG channels, particularly in the frontal, parietal, and temporal, than the HC groups. The delta power band on the source level was increased for the AD group and decreased for the HC group. In contrast, the source activities of alpha, beta, and gamma frequency bands were significantly reduced in the AD group, with a high decrease in the beta frequency band in all brain areas. Furthermore, the coherence of rEEG among different EEG electrodes was analyzed in the beta frequency band. It showed that pair‐wise coherence between different brain areas in the AD group is remarkably increased in the ECR state and decreased after subtracting out the EOR state.

**Conclusion:**

The findings suggest that examining PSD and functional connectivity through coherence analysis could serve as a promising and comprehensive approach to differentiate individuals with AD from normal, which may benefit our understanding of the disease.